# Pathways to the development of purpose in life among ethnic minority adolescents

**DOI:** 10.1111/jora.70299

**Published:** 2026-10-16

**Authors:** Rosa Kim, Meeyoung O. Min, Sarah L. Canham, Morgan Williams

**Affiliations:** ^1^ College of Social Work, University of Utah Salt Lake City Utah USA

**Keywords:** adolescents, ethnic minority, growth mindset, pathways, purpose in life

## Abstract

Purpose in life has been defined as a sense of meaning, direction, and commitment to valued long‐term goals. Developing a sense of purpose in life is an important task during adolescence and considered an essential developmental asset. However, current understandings of how adolescents, particularly those with an ethnic minority background, develop their life purpose remain limited. This qualitative study explored the pathways through which ethnic minority adolescents develop a sense of purpose in life. This study conducted 19 semi‐structured interviews with ethnic minority adolescents, mainly Hispanic and Asian, aged 14–18 years old (47% male) residing in urban areas of the Mountain West region of the United States. Interviews were conducted in person at a location preferred by participants (63%) or via Zoom between December 2023 and April 2024. Data were analyzed using a thematic analysis approach. Ethnic minority adolescents described four pathways to developing their sense of purpose in life: proactive (through active exploration and curiosity), reactive (in response to a meaningful or transformative life event), social learning (through the observation and modeling of others), and growth mindset (through the belief in personal growth from challenges). Our findings align with three pathways previously theorized in the literature (proactive, reactive, social learning) and suggest growth mindset as an additional pathway. The growth‐mindset pathway appeared to be particularly relevant for ethnic minority adolescents, as they often navigate adverse life experiences within the broader majority culture. This study contributes to our understanding of how ethnic minority adolescents can transform adverse life experiences into opportunities for developing a sense of purpose in life.

## INTRODUCTION

Purpose in life has been defined as “a central, self‐organizing life aim” (Kashdan & McKnight, 2009, p. 304) and how people perceive their lives as having meaning, direction, and goals (Kim et al., 2022; Ryff, 2014; Steger et al., 2006). It is demonstrated through setting clear personal goals, holding beliefs that bring life meaning, and having a significant sense of being (Ryff & Singer, 1998). Having purpose in life is positively associated with an individual's psychological well‐being throughout both adolescence and adulthood (Bronk, 2011; Hill et al., 2010). In particular, establishing a sense of purpose in life is a key developmental task (Benson et al., 2011; Bronk et al., 2009; Damon et al., 2003) and plays a vital role in promoting youth thriving, flourishing, and positive development (Bronk, 2012; Damon et al., 2003; Hill et al., 2010). Numerous studies have documented that adolescents with a sense of life purpose are more likely to establish healthy life goals, which can help them overcome a wide range of psychological challenges (Kim et al., 2014; Schippers & Ziegler, 2019) as well as become more resilient to difficulties, pressure, and strain (Kashdan & McKnight, 2009).

During adolescence, young people begin exploring and understanding their self and determining the core values they wish to hold, while actively forming their identities through interactions within their multidimensional environmental contexts (e.g., ecological, sociocultural, historical, and sociopolitical) (Erikson, 1968; Olneck, 2004). The formation of a stable and concrete sense of personal identity, which is closely tied to one's perception of purpose in life (Heine et al., 2006; Steger et al., 2009), has a powerful influence on establishing a sense of purpose in life (Negru‐Subtirica et al., 2016; Newman et al., 2019; Sumner et al., 2015). A person's concrete self‐identity can also position adolescents to move forward to the next stage of exploring and developing their purpose in life (Kiang, 2012).

A sense of purpose in life is understood to develop during adolescence (Fry, 1998; Steger et al., 2009) as adolescents start to explore life goals and reflect on what makes life meaningful (Malin et al., 2014). Individuals who understand what gives their life purpose are better equipped to answer the question of who they are by articulating their own goals and desires (Hill et al., 2013). As an essential human drive, finding purpose in life can be especially important during adolescence (Burrow et al., 2010), since having purpose in life serves as a significant facilitator that provides motivation and guides youth toward positive developmental pathways (Bronk et al., 2010).

### Pathways to developing purpose in life

The process of purpose development is not always straightforward or predictable (Newman et al., 2019), as it often involves recognizing deeply held personal values, overarching aims, and pursuits that are believed to foster personal growth and bring a deep sense of fulfillment (Kosine et al., 2008). A sense of purpose in life can be developed by personal attributes, significant life changes, identity formation processes, social supports and influences, and a sense of belonging within the community (Hill et al., 2016; Malin et al., 2014; Newman et al., 2019). Influenced by unique experiences and life contexts, adolescents may navigate their journey of searching for and developing a sense of purpose in life through diverse pathways.

Although fully capturing all possible pathways to the development of purpose in life can be challenging, Kashdan and McKnight (2009) proposed three potential pathways: proactive, reactive, and social learning. The proactive pathway is characterized by continued effort over time, with a sense of purpose emerging gradually through ongoing modification and clarification. Within this pathway, individuals need to be open and receptive to new experiences and alternative ways of understanding themselves and their surroundings (Bishop et al., 2004; Kashdan & McKnight, 2009; McCrae, 1993). Another pathway is the reactive pathway, which is driven by a transformative life event that brings about a new sense of purpose in life and clarity. A key aspect of the reactive pathway is that a significant life event triggers individuals to explore different behaviors and possibilities through trial and error, ultimately contributing to the development of a clearer sense of purpose (Kashdan & McKnight, 2009). Lastly, the social learning pathway involves having one's purpose in life shaped through observing, imitating, and modeling others (Hill et al., 2014; Kashdan & McKnight, 2009). In this pathway, purpose emerged through observing others' behaviors and experiencing an emotional response to those behaviors (Kashdan & McKnight, 2009).

Despite the importance of understanding pathways to purpose development, few empirical studies have examined whether the three theorized pathways are supported by evidence or whether they represent all possible pathways to purpose development. Though Hill et al. (2014) examined the personality and well‐being correlates of the three distinctive (proactive, reactive, and social learning) pathways, this study utilized a sample of predominantly White individuals in young to middle adulthood without exploring additional potential pathways.

### Ethnic minority adolescents and the development of purpose in life

Given that adolescents come from diverse backgrounds and possess unique life experiences and that development of purpose in life is highly context‐dependent (Liang et al., 2017), empirical research involving diverse adolescents is critical to enhancing our understanding of youth's life purpose (Hill et al., 2013; Kashdan & McKnight, 2009). While experiencing a wide range of stressors and challenges is relatively common during adolescence due to rapid developmental changes across physical, psychological, and social domains (Hellström et al., 2024; Romeo, 2013), adolescents from ethnic minority backgrounds often encounter additional sources of adversities, including exclusion, discrimination, and marginalization rooted in their racial, ethnic, social, and cultural backgrounds (Kim et al., 2025). These experiences are particularly salient within the racialized and stratified nature of U.S. society, where race‐ and ethnicity‐based discrimination and marginalization remains a common experience among many ethnic minority adolescents (Benner, 2017). Although these contextual challenges can be considered as stressful life experiences, they can also inspire and shape one's purpose development (Zhou et al., 2023), as a sense of purpose in life can sometimes emerge from adverse experiences (Malin et al., 2019).

The meaning‐making model (Park & Folkman, 1997), widely applied in research on stress, coping, and posttraumatic growth, can offer a useful framework for understanding how ethnic minority adolescents develop a sense of purpose in life from contextual life challenges and provides complementary perspectives to Kashdan and McKnight's (2009) three potential pathways. This model describes meaning‐making as the process of interpreting adverse or highly stressful life events and connecting them to a broader system of meaning that encompasses one's beliefs, goals, and life purpose (Park & Ai, 2006); and this meaning‐making process, along with reframing life goals following adversity, can also foster the development of purpose (Malin et al., 2019). Extant research on adversity and stress indicates that making meaning of challenging circumstances is a coping mechanism that can foster psychological growth (Kiang & Fuligni, 2010; Schaefer & Moos, 1992). Through meaning‐making processes, adolescents from ethnic minority backgrounds may transform challenging experiences into opportunities for purpose development. From this perspective, ethnic minority adolescents' interpretations of experiences such as exclusion and marginalization within a majority society may play a central role in shaping the meaning they make and, in turn, their sense of purpose. This suggests that ethnic minority adolescents' pathways to purpose development may not be fully captured by the theorized known pathways and may instead reflect additional or distinct pathways.

### Aims of the current study

Although the extant literature has discussed the development of purpose in life among adolescents, few studies have focused on identifying the pathways through which purpose develops (Malin et al., 2014). In addition, to the best of our knowledge, no existing research has empirically explored these pathways among ethnic minority adolescents. Grounded in Kashdan and McKnight's (2009) theoretical work and Park and Folkman's (1997) meaning‐making model, this study explored how ethnic minority adolescents develop a sense of purpose in life through reflecting on and making meaning of their lived experiences. Specifically, this study sought to better understand the process through which purpose develops among ethnic minority adolescents. To address this aim, the study was guided by two research questions: (1) How do ethnic minority adolescents find and develop a sense of purpose in life? and (2) Do ethnic minority adolescents exhibit distinct pathways to purpose development beyond the three established pathways?

## METHODS

### Procedures

This qualitative exploration was conducted following a survey that examined ethnic minority adolescents' sense of purpose in life, culture, and behaviors. We conducted in‐depth, semi‐structured interviews with a subset of survey participants to explore how ethnic minority adolescents develop their purpose in life, centered around participants' meanings and development of purpose. First, survey participants who identified as an English‐speaking ethnic minority aged 14–18 were recruited from two local youth‐serving agencies, a university‐sponsored public event for minority high school students, and through snowball sampling in which participants were encouraged to recruit their friends. Following the survey, purposeful sampling was used to recruit interview participants.

The final survey question asked participants to indicate whether they consented to be contacted for a follow‐up interview. A graduate Master of Social Work (MSW) student contacted potential interview participants who had consented to follow‐up contact and scored at or above the 75th percentile on the Claremont Purpose Scale (CPS; Bronk et al., 2018), which assesses three dimensions of purpose: meaningfulness, goal orientation, and beyond‐the‐self. Because higher CPS scores indicate a greater overall sense of purpose and the presence of most or all dimensions of purpose (Bronk et al., 2018), participants scoring at or above the 75th percentile were selected for interviews, as they were expected to provide richer, more nuanced, and more clearly articulated perspectives on their sense of purpose.

A subsample of 26 survey respondents was invited to an interview, and 19 agreed to participate. Parental consent and adolescent assent were obtained via either paper‐based or online form prior to study participation. After consent and assent were received, a parent or legal guardian was contacted to verify study participation only for adolescents who submitted assent and consent forms online, using the contact information provided. This study was approved by a University Institutional Review Board, and participant identifiers have been removed to de‐identify participants.

### Participants

Participants included nine males and 10 females, with a mean age of 15.6 (SD = 1.38) years (Table 1). All participants were in middle or high school, except one who was 18 years old and a college freshman. Seventeen participants (89.5%) were born in the U.S., and two were born outside of the U.S. (Costa Rica, South Korea). Over half of the participants self‐identified as Hispanic (*n* = 12, 63.2%), and the rest self‐identified as Asian (*n* = 6) or African American (*n* = 1).

**TABLE 1:** Demographic characteristics of participants.

Participants	Age	Grade	Sex	Ethnicity	Place of birth
1	14	9th	Male	Hispanic	U.S.
2	17	12th	Male	Hispanic	U.S.
3	17	12th	Female	Hispanic	U.S.
4	14	8th	Male	Hispanic	U.S.
5	15	10th	Female	Hispanic	Costa Rica
6	18	12th	Female	Hispanic	U.S.
7	16	10th	Female	Hispanic	U.S.
8	15	10th	Male	Hispanic	U.S.
9	14	9th	Female	Asian	U.S.
10	17	12th	Female	Hispanic	U.S.
11	16	10th	Female	African American	U.S.
12	16	11th	Male	Hispanic	U.S.
13	15	9th	Female	Asian	U.S.
14	17	10th	Male	Asian	U.S.
15	14	9th	Female	Hispanic	U.S.
16	14	9th	Female	Hispanic	U.S.
17	15	10th	Male	Asian	South Korea
18	15	10th	Male	Asian	U.S.
19	18	Freshman (College)	Male	Asian	U.S.

### Data collection

All interviews were conducted by an MSW student interviewer trained in qualitative data collection methods between December 2023 and May 2024 at a time and location convenient and comfortable to participants. Before conducting the interview, the interviewer explained the purpose of the study to the participant and received permission to audio record the interview. Twelve interviews were conducted in person at local libraries and community organization offices, and seven were conducted virtually via Zoom. The semi‐structured interview agenda (Appendix S1) asked participants about the presence of purpose in their life, their goal orientation, processes through which their life purpose developed, and ideas about their future. The interviews were transcribed using Otter.ai, a transcribing software, or through the Zoom audio transcription function. All transcripts were assigned alphanumeric codes to maintain participant anonymity and were reviewed by the interviewer to ensure accuracy. Interviews lasted 15–20 min, and detailed field notes were taken during each interview. During the weekly debrief meeting, the research team reviewed field notes and discussed the interviews that were shorter than originally planned. The identified factors, including creating a more comfortable interview environment, encouraging participants to reflect more deeply on their experiences, and using strategies to address participants' tendency to provide brief answers, were taken into consideration during subsequent interviews. Participants received a $40 gift card as an honorarium for their time and participation.

### Data analysis

We employed a thematic analysis approach (Braun & Clarke, 2006, 2022) using the qualitative data analysis software NVivo 14 (QSR International, 2023) to enable systematic coding and data organization. The first and third authors first familiarized themselves with the data by reading and re‐reading the transcripts. Through the iterative process of reading to understand the data, we noted similar themes that were identified in prior research on adolescents' development of a sense of purpose in life (Hill et al., 2014; Kashdan & McKnight, 2009; McKnight & Kashdan, 2009) and generated initial low‐level codes. Guided by Kashdan and McKnight's (2009) conceptual framework, we initially examined whether the previously identified pathways (proactive, reactive, and social learning) were reflected in the data. In building our codebook, we used terms from prior research and theory (deductive coding) while also coding text as the data suggested variations on existing frameworks of adolescents' purpose in life (inductive coding). Themes were then developed and prioritized based on their prevalence, depth, and relevance across participants' narratives (Braun & Clarke, 2006). Although alternative and divergent themes were discussed throughout the analytic process, only themes supported by sufficient evidence were retained. Following codebook development, we reviewed and defined our themes (Braun & Clarke, 2006, 2022). Through ongoing team discussions, we examined whether and how the themes we identified adequately captured adolescents' pathways to developing their purpose in life and modified themes and codes, as needed, to reach consensus. To enhance analytic rigor, memos were written throughout the analysis to document the analysts' interpretations and decisions (Birks et al., 2008).

### Researcher positionality

The authors of the current study include two PhD‐trained scholars, one PhD student, and one MSW student. The lead author is an ethnic minority immigrant, mother, and international PhD student in social work in the U.S. whose lived experiences informed her commitment to understanding and supporting immigrant youth and families. The second author, also a former international student and parent of two minority emerging adults, brings expertise in youth development among youth experiencing adversity. The third author brings qualitative expertise but has little prior knowledge of research into the purpose in life or adolescence; their analysis and interpretation of the data were influenced by their lifelong residency in various cities across North America, which limits their understanding of the experiences of children from immigrant families in the U.S. The last author, an MSW student, conducted interviews and brought her experience with working with diverse youth populations. She identifies as White and approached this research with a recognition of her own privilege and social positioning and aimed to center participant experiences throughout the interview process. Given that reflexive examination of researchers' values, biases, and positionality is central to both data collection and analysis (Tracy, 2010), we acknowledge that each researcher's backgrounds, experiences, and perspectives may influence the research process and interpretation of findings. The authors aimed to center the voices and lived experiences of ethnic minority adolescents, explore factors that support their positive development, and critically examine potential social and structural factors that may shape experiences of marginalization. Throughout the study, the authors engaged in ongoing critical reflection on the difference between themselves and the participants (Segall et al., 1998) and sought to minimize adult‐centric interpretations by centering adolescents' perspectives and lived experiences.

## FINDINGS

Using prior research to guide our understanding of how adolescents develop their purpose in life (Hill et al., 2014; Kashdan & McKnight, 2009; McKnight & Kashdan, 2009), we first organized the data into three pathways that have been previously described: proactive, reactive, and social learning. Additionally, we found that adolescents also developed their sense of purpose in life through a growth‐mindset pathway, in which they viewed challenges and setbacks as opportunities for growth and integrated these experiences into their purpose development. This pathway appears to be distinct from previously theorized pathways, particularly the reactive pathway, as discussed in detail below.

### Proactive pathway to developing purpose in life

Having a proactive pathway to developing one's purpose in life suggests that individuals actively search for their potential life goals by fostering curiosity and exploring new experiences (Hill et al., 2014; Kashdan & McKnight, 2009). Given the continuously evolving developmental stage of adolescence, most of our participants identified that they were actively exploring and developing their lives' purposes. Though some participants were unsure about their purpose in life, they identified possible life goals, including goals to pursue new experiences. Participants also described that they are still trying to determine what they should do and what their life purpose is. One male participant (P19, 18‐year‐old) stated:

I feel like my purpose right now is just to, funny enough, find my purpose. I really feel like, because I'm still growing and still trying to figure out the ‐ what the works of life, just to really see, venture out, explore different opportunities, and to see what really I find myself enjoying the most, and then to commit to that enjoyment that I have…I feel like I do enjoy learning. And I feel like we're learning somewhat does give me a purpose. And I feel, specifically more about the medical field. I am very interested in that whole area and being able to see the different areas of the medical field and…how each one offers different things to the people who participate in it. I feel like being able to observe all these different fields, and then finding one that suits me the best, and pursuing that is my purpose.

P19 had explored different activities, responsibilities, and experiences and expected to learn from them in order to find his purpose in life. Another male participant (P14, 17‐year‐old), who indicated he had not considered his purpose in life prior to the interview, described the ways in which he was seeking to discover it, aiming to achieve happiness and fulfillment:

I haven't really thought about my purpose in life too much, but I just really want to keep it simple where I want to find my happiness. And even if I have to do things that doesn't really necessarily bring me happiness, I feel like to do those things, I need something that brings me joy to help motivate me, and what I am doing.

A female participant (P13, 15‐year‐old) described her pathway to developing a purpose in life as being closely related to living a life connected to others while having new and different experiences and accomplishments:

I think in life you have a lot of aspects that can give you purpose, but sometimes not all of them will work out. So, the actual purpose in your life is to just experience stuff and then fulfill them, or just work hard for it, and the giving and putting out love part, I think that's kind of my purpose in life is to show love, so that other people can also have a purpose in life. It's just like a mutual kind of thing.

Varied activities (e.g., playing video games, sports, school clubs) were also identified as helpful to participants proactively finding direction and purpose in life. One male participant (P1, 14‐year‐old), who aspires to become a lawyer, mentioned that a video game in which he plays a lawyer helped him develop his life purpose. He described learning a lot from practicing law within the game while enjoying the experience of playing the character of a lawyer:

The one game I did is a game about being a lawyer—what they do, and how they defend people in court. And that was the only game I found interesting and fun… As the game gets progressively harder, I have to retry and retry, think of different solutions I could do to defend my person…and I learn a lot from it.

One male participant (P17, 15‐year‐old), who was still trying to find his sense of purpose in life, shared that he had been participating in diverse school clubs because he felt they helped him explore his future direction and develop a sense of purpose. He stated, “I've found myself doing stuff like a bunch of clubs that are shaped for my future…they show you what you're supposed to do.” A female participant (P13, 15‐year‐old), who described sports as an important source of life purpose, stated, “For my sport, I had some roller coaster moments where I had injuries and stuff. But I did achieve a lot in that sport, and I've been kind of taking a break and trying different sports in school.” Consistent with adolescents' developmental stage of self‐exploration and real‐word engagement (Saragosa‐Harris et al., 2022), participants appeared to actively pursue diverse, interest‐driven experiences in the process of discovering and developing their purpose in life.

### Reactive pathway to developing purpose in life

Another pathway to developing one's life purpose is the reactive pathway, where a sense of purpose in life develops in response to transformative life experiences or significant life changes or events (Hill et al., 2014; Kashdan & McKnight, 2009; McKnight & Kashdan, 2009). Adolescent participants identified specific life events or experiences, such as volunteering, the COVID‐19 pandemic, or family/peer‐related life events that influenced the development of their purpose in life. One female participant (P9, 14‐year‐old) shared that her volunteer experience influenced her life purpose development:

A lot of it came from just my experiences with doing a lot of service work. I think that was one of the times where I felt that was really like real purpose, especially when I would do it with other people, it just felt more purposeful to achieve something good with other people.

Similarly, a male participant (P4, 14‐year‐old) described developing his life purpose—to encourage more people to get involved in community advocacy—through recognizing that people in his local community were not aware of important events or issues. He stated, “If we get more families to come to these events [community development meetings], we could get a lot of more people into the community.”

Several participants also reported that the COVID‐19 pandemic influenced the development of their purpose in life. One male participant (P17, 15‐year‐old) shared that his current life purpose is to do his best in school because of the disruption experienced during and after the pandemic.

The year after COVID—because during COVID, I really didn't care enough about school cause everything's online anyways—went back to school, I feel I really got struck. It was a little crisis, because I don't know what I'm doing here anymore. And like, I didn't keep up the year before. I'm kind of behind in a way.

Another male participant (P18, 15‐year‐old), who identified his sense of life purpose as fostering a joyful environment for his family and others, recognized this purpose because of the pandemic.

As soon as I got back in school [after the pandemic], around the freshman year of high school, that was when people started to actually be able to communicate with each other once again in person, because in middle school I was a kid who was always online. So, I didn't have that interpersonal connection with people at my school. I think around that time was when I realized that I needed those connections, those human connections…in order for me to actually kind of be me again. I kind of lost what that feeling was during the pandemic, so, being able to communicate once again at the end of it was a really big awakening for me.

Given that the COVID‐19 pandemic significantly impacted individuals' lives, it became a significant life event that compelled adolescents to reflect on their life purpose.

Family‐ and peer‐related life events also appeared to influence adolescents' sense of purpose development. One female participant (P10, 17‐year‐old), who identified her sense of purpose as helping others and making someone's day better, shared that she had a family member who had an accident and was hospitalized, which led her to discover a healthcare‐related program at school and influenced her future direction: “I started noticing that school had a program that led me into the medical field…their main job is to support others and make sure they feel comfortable.” Another female participant (P9, 14‐year‐old), who described her parents and family as religious, mentioned that she was able to develop her purpose in life because of the negative influences from a friend:

I had a hard time with a friend, and this friend that was going through a really hard time. And there's just a lot of negativities around me, which made me feel really like hopeless…I think once I actually started thinking about it, I was able to feel like hope, and I felt a lot better…It was this time where this one friend of mine she was doing self‐harm along with a few other people who were friends with her. So there really was no positive input.

Although most participants who developed their purpose in life through the reactive pathway did not report extreme life‐changing events, they clearly identified experiences that shaped their sense of purpose in life through reflection and positive reinterpretation of meaningful life events and turning points.

### Social learning pathway to developing purpose in life

Participants described ways in which they developed their purpose in life by observing and learning from social and cultural influences. A female participant (P12, 16‐year‐old) mentioned that her purpose in life was closely related to her culture:

Purpose is something you want to see before you die or to live the life that your culture has set for you…I'm Hispanic, so mainly for the girls, they're kind of saying you should have a Quinceañera [celebration of a girl's 15th birthday] and then after that…ready for marriage…then you're set to like have a job, have a family, and then kind of provide for your family.

Adolescent participants also developed their purpose by observing values and beliefs passed down by their family. A female participant (P10, 17‐year‐old), who developed her purpose through observation of her parents, mentioned, “My parents are very caring in helping others. My parents have always just made separate time to go in the church. So, I've kind of gotten that from them.” A male participant (P19, 18‐year‐old) also learned from his family and stated that his family has been influential in developing his purpose in life related to the medical field:

I feel like family is definitely a thing to consider, because I had people close to me that either had medical issues or were related to the medical field. So just seeing them act in their natural capacity and being able to observe it from a third person, made me more interested in and getting involved in that certain career course and profession.

A female participant (P9, 14‐year‐old) similarly stated that she developed her life purpose related to school while watching her mother:

I've always known school is very important to me. My mom is a teacher, and I've seen how she's achieved a lot from being able to be educated…this year where I finally understood for myself why it was important and why I was doing it.

Another female participant (P7, 16‐year‐old) also developed her sense of life purpose related to school while observing her close family members:

I have a lot of family members. They didn't finish up like school or anything. So that really did impact them. That made me and motivated me to prove them. Like, they would be proud of me. I would want to achieve that.

While some adolescents developed their purpose in life by observing family members, others did so through transmission from their family. A female participant (P9, 14‐year‐old), whose life purpose is to be remembered as a good person like her grandparents, described:

I've always wanted to be my great grandparents, or just my grandparents, too. I always hear really good stories about them, things that they did good. I don't know, but I've always wanted to kind of be that person because I also kind of don't want to be like forgotten in the future.

Another female participant (P11, 16‐year‐old), who values education as her life purpose, mentioned that she was taught this from her parents:

It's been a huge part of my life. My parents have always taught you can't go anywhere without an education, or…they know people who didn't go to school and didn't graduate high school, and they're not doing very well life right now.

For participants who developed their purpose in life through the social learning pathway, people in their immediate social network, particularly family, played a crucial role as participants observed and learned from those around them and their culture.

### Growth‐mindset pathway to developing purpose in life

The final pathway newly identified in this study is termed the growth‐mindset pathway. The growth mindset refers to the belief that human abilities are not fixed and can be developed across the lifespan through effort, practice, and education (Dweck, 2016; Dweck & Yeager, 2019). The idea of growth mindset aligns with the meaning‐making model, as both involve reinterpreting a situation in new ways and reassessing one's beliefs and goals to reconstruct a cohesive understanding among them (Park & Ai, 2006). Several studies have also noted that individuals with a growth mindset tend to evaluate themselves accurately, trust in their intelligence and abilities, willingly confront challenges, and continue to grow through their failures and setbacks (Dweck, 2016; Zhao et al., 2023). These characteristics demonstrate how a growth mindset can shape the ways individuals construct meaning from their life experiences (Zhao et al., 2023), supporting adolescents' purpose development through growth‐oriented interpretation of stressful life events.

Participants with a growth mindset demonstrated that their pathway to the development of purpose in life is shaped by the positive belief in their ability to grow, improve, or overcome difficulties. A female participant (P9, 14‐year‐old) who strives to maintain a positive perspective while developing her life purpose mentioned:

I think it was last year I had a really negative mindset. I think it's especially harder to get out of thinking like that, and it really affects the things that I do. So, when I started trying to have more like positive mindset and thinking more broader perspective of things that I was doing…Maybe it changes the things that you do.

Another female participant (P13, 15‐year‐old), who reported that making persistent efforts toward her life purpose is important, said:

I'm kind of self‐motivated…I want to have a good career, and even if things don't work out, I want to at least try like 100%…If the results aren't always good, it feels better to say that you at least put in all your effort.

Several participants who described that their purpose in life is to achieve academic success and go to college also demonstrated a growth mindset. One male participant (P14, 17‐year‐old) stated:

I feel like for many people, including me, puts a lot of pressure on me, so I try not to think about it too much. Just think about all the things in my daily life and think about the small things. And sometimes I just look at the big picture to help me visualize what I need to do right now… I think that really motivated me to continue what I'm doing.

Another female participant (P9, 14‐year‐old), whose sense of purpose was closely tied to academic achievement, explained that her siblings had always performed better academically than she had. However, she mentioned, “People who might be super good at math might not be as good as something that I might be good at”, which demonstrates her ability to focus on her own growth and strengths. Similarly, another female participant (P15, 14‐year‐old), who described negative experiences with teachers suggested that she worked to succeed despite these experiences:

I've had some teachers that just don't like me or make it really hard to succeed in their class… usually what I'll try to do is to do really well in that class. So, even if they don't like me or it's like they're trying to not make me succeed in their class, I can still do the best that like I can do, even without their support.

Participants who demonstrated a growth‐mindset pathway to developing purpose in life tended to embrace and reframe challenges and describe optimism about negative experiences and struggles by practicing positive thinking and transforming negatives into motivators for personal growth.

## DISCUSSION

Finding and developing a sense of purpose in life has been regarded as a fundamental human motivation and has a crucial role in promoting positive development during adolescence. While previous studies have highlighted the importance of finding purpose in life, less attention has focused on ethnic minority adolescents' purpose development. By thematically analyzing in‐depth interviews, the present study identified pathways through which ethnic minority adolescents develop a sense of purpose in life.

Consistent with previous research, proactive, reactive, and social learning pathways to developing purpose in life (Hill et al., 2014; Kashdan & McKnight, 2009) were found, and one additional pathway, growth mindset, was identified in this study. Findings suggest that adolescents following a proactive pathway actively seek and engage in new experiences that match their interests, supporting their efforts to discover and develop their sense of purpose. This corroborates previous research suggesting that individuals who take a proactive pathway tend to show greater openness to new experiences (Hill et al., 2014). Increased exploration, a normative characteristic of adolescent development (Saragosa‐Harris et al., 2022), may further contribute to adolescents' proactive pathway toward developing a sense of purpose in life, as adolescents become more interested in exploring and engaging in their surroundings. Additionally, families and schools can support adolescents in developing a sense of purpose in life through the proactive pathway by encouraging them to explore their interests and fostering exploration and creativity.

In contrast, participants who followed a reactive pathway developed their purpose in life in response to, or through, meaningful and transformative life experiences. While existing literature indicates that transformative life events, whether through direct or indirect exposure to trauma, can lead individuals to reflect on or begin forming a sense of purpose in life (Kashdan & McKnight, 2009; Tedeschi & Calhoun, 2004), adolescents in this study did not explicitly describe their experiences as traumatic. However, they identified specific life events or personal experiences, such as volunteering, the COVID‐19 pandemic, or family‐ and peer‐related situations, as influential in shaping their life purpose. Given that the reactive pathway allows adolescents to reflect on specific life events and synthesize these experiences to develop a sense of purpose in life (Kashdan & McKnight, 2009), helping them identify and reflect on their experiences may deepen their understanding and foster the development of purpose in life.

The social learning pathway was also found to guide adolescents in developing their sense of purpose in life as they observed and modeled the behaviors and values of people in their environment, as described in prior research (Kashdan & McKnight, 2009). For instance, cultural and family values, whether practiced or observed, played a role in shaping adolescents' purpose development. Participants described observing prosocial behaviors of others in their environment, such as caring for others and valuing education, and reflecting these in their reports of their life purpose. This pathway differed from proactive and reactive pathways, as adolescents developed their sense of purpose in life by learning from others, rather than through active exploration or reaction to life events. Given that prior research has noted that individuals may come to understand the value of having purpose in life and potentially develop their own life purpose through social learning (Hill et al., 2014), it is important to underscore the potential impact of supportive adults or role models who can help adolescents navigate their life experiences, cultivate a sense of life purpose, and learn how to live out that purpose in life.

Compared with the proactive and reactive pathways, purpose development through the social learning pathway appeared to be more strongly shaped by participants' social contexts, particularly their family, cultural, and ethnic backgrounds. For instance, elements of participants' social contexts were not explicitly discussed in reflections on the proactive or reactive pathways. These findings may suggest that ethnic minority adolescents follow the purpose development pathways previously identified in the literature; however, ethnic and cultural backgrounds may be particularly salient within the social learning pathway.

Finally, the growth‐mindset pathway was identified in the present study as ethnic minority adolescents described that their life purpose was developed from their belief in their ability to grow, improve, or overcome difficulties. For instance, adolescents who experienced academic challenges or negative school experiences used these struggles as opportunities for personal growth through effort and practice. Adolescents in this study appeared to interpret and approach similar challenges or situations through a growth mindset, connecting these experiences in ways that strengthen their life purpose development.

Given that a growth mindset is not an inborn trait (Dweck, 2016), these findings highlight the need to further examine how adolescents develop this mindset and whether it should be considered as a learned behavior. The present study also suggests that the growth‐mindset pathway reflects how adolescents perceive and interpret challenges as opportunities for growth, which appears distinct from the proactive pathway that is driven by curiosity, self‐motivation, and self‐exploration and the reactive pathway that involves reflection and adaptation based on personal experiences. However, questions remain about how the growth‐mindset pathway meaningfully differs from the reactive pathway. In the present study, the growth‐mindset pathway was identified as a distinct pathway rather than a subtheme of the reactive pathway, as the reactive pathway did not fully capture the nuances reflected in the growth‐mindset pathway. This distinction is supported by participant reports that demonstrated their active engagement in striving and personal growth following specific experiences and their intentional integration of this growth into their development of life purpose. Therefore, the growth‐mindset pathway appears to be a distinct pathway, as adolescents may foster their life purpose through growth that follows from their specific life experiences.

The growth‐mindset pathway may be more relevant to ethnic minority adolescents as they are more likely to encounter adverse life experiences that are beyond their control, including racial/ethnic discrimination, exclusion, and marginalization (Benner, 2017; Umaña‐Taylor & Rivas‐Drake, 2021). While challenges and difficulties are common during adolescence, not all adolescents interpret adversities as opportunities for growth. The experience of marginalization may, instead, undermine motivation, constrain learning and adaptation, and narrow adolescents' perceptions of future possibilities (Sumner et al., 2018). Considering ethnic minority adolescents' position within a majority society, the challenges they frequently encounter may require them to navigate, adapt, and make sense of adverse experiences more frequently and actively than their nonminoritized peers. And when adolescents successfully navigate these challenges, they may develop a strong belief in their capacity to learn and grow through adversity. Such experiences may strengthen their sense of self and self‐efficacy, making the growth mindset particularly relevant to ethnic minority adolescents' purpose development. As the growth‐mindset pathway was newly conceptualized in this present study based on data from ethnic minority adolescents, it requires further validation across other samples of diverse adolescents to establish its broader applicability.

### Strengths and limitations

A salient strength of this study is its contribution to identifying a potential new pathway to purpose development among adolescents, the growth‐mindset pathway. This offers a more nuanced understanding of purpose development among ethnic minority adolescents by highlighting how they may develop a sense of purpose in life through interpreting challenges and setbacks as opportunities to grow. In addition, we believe the current study is one of the first to provide empirical evidence of the three theorized pathways (proactive, reactive, and social learning) among ethnic minority adolescents. Our study addresses a literature gap where previous research has predominantly utilized relatively privileged, racially and ethnically homogeneous populations (e.g., Hill et al., 2014) and suggests that these established pathways may also be applicable to more diverse populations, including ethnic minority adolescents. Taken together, the previously established pathways and the newly identified growth‐mindset pathway provide a more comprehensive understanding of how ethnic minority adolescents' sense of purpose in life can be fostered and developed through their lived experiences. These findings have implications for both theory and practice and may help inform interventions designed to support purpose development among diverse adolescent populations.

There are some limitations that should be considered. First, the duration of most interviews was shorter than expected. This may relate to the abstract concept of purpose in life, which adolescents may find difficult to conceptualize or verbalize due to the ongoing development of their abstract thinking abilities (Christie & Viner, 2005). Although adolescent participants were able to share meaningful insights, the relatively short interview duration might not have captured the nuanced aspects of participants' experiences. Future research may benefit from sharing questions with interviewees prior to the interview, allowing them the opportunity to reflect and develop more comprehensive narratives (Haukås & Tishakov, 2024). Second, interviews were conducted either in person or via Zoom, depending on the preference of each participant. Although both interview modes were found to offer similar levels of support and conversational engagement, and virtual interviews increased accessibility for adolescent participants, the quality of the data may vary based on the degree of privacy in the interview setting (Anthony et al., 2025). Third, adolescents may develop their life purpose through a combination of multiple pathways (Kashdan & McKnight, 2009), and approximately one third of participants described developing a sense of purpose in life through a combination of multiple pathways in this study. Although the present study focused on identifying whether previously established pathways were evident among ethnic minority adolescents and whether any additional distinctive pathways emerged in this population, future research should examine whether adolescents engage in a single or multiple pathways to purpose development simultaneously or/and sequentially and how these multiple pathways may reciprocally influence one another. Fourth, given the relatively small sample size, the current study was unable to meaningfully examine potential differences across developmental stages, ethnic/racial backgrounds, and immigrant‐related experiences. As our study aimed to explore the experiences of ethnic minority adolescents' experiences in developing a sense of purpose in life rather than comparing across demographic subgroups, the findings may not fully capture how contextual factors influence participants' experiences. Future research should investigate these potential differences in greater depth. Lastly, findings cannot be generalized to other racial and ethnic minority adolescents, as study participants were primarily Hispanic or Asian adolescents. Differences may exist between racial and ethnic groups; future research should aim to identify unique influences on the development of purpose in life using other ethnic minority adolescents.

## CONCLUSION

The present study provides valuable insights into the various pathways through which ethnic minority adolescents develop their sense of purpose in life. Ethnic minority adolescents develop their life purpose proactively, reactively, by learning from people in their environments, and by reframing challenges as opportunities for growth, all highlighting the importance of providing opportunities, support, and guidance as they navigate purpose development. Future studies that explore whether ethnic minority adolescents' unique experiences stemming from their ethnic and cultural backgrounds influence their growth mindset and whether this mindset functions as a mechanism for resilience and adaptability will enhance our understanding of the growth‐mindset pathway.

## AUTHOR CONTRIBUTIONS

**Rosa Kim:** Conceptualization; writing – original draft; methodology; writing – review and editing; formal analysis. **Meeyoung O. Min:** Conceptualization; writing – review and editing; funding acquisition. **Sarah L. Canham:** Methodology; writing – review and editing. **Morgan Williams:** Writing – review and editing; project administration.

## FUNDING INFORMATION

This research was supported by the University of Utah VPR Research Incentive Seed Grant.

## CONFLICT OF INTEREST STATEMENT

None.

## ETHICS STATEMENT

The ethical approval for this study was approved by the Institutional Review Board of the University of Utah. Written informed consent from parents and assent from adolescents were obtained from all participants at the start of the study.

## CONSENT

All adolescent participants provided assent, and written informed consent was obtained from their parents or legal guardians prior to participation.

## Supporting information


**Appendix S1.** Youth purpose qualitative interview questions.

## Data Availability

The data that support the findings of this study are available on request from the corresponding author. The data are not publicly available due to privacy or ethical restrictions.
