# Supplementary material for: Pathways to the development of purpose in life among ethnic minority adolescents
Source: J Res Adolesc. 2026 Oct 16;36(4):e70299. doi: 10.1111/jora.70299 (PMC13675483; doi:10.1111/jora.70299)
Supplement: Supplementary file 1 [file JORA-36-0-s001.docx]

**Appendix A. Youth Purpose Qualitative Interview Questions**

**Introductory/opening statements & questions**

- What motivated you to participate in the study (other than the $40 gift card)? [If the gift card was indeed only motivation, ask why getting $40 was important?]

1. **The presence of purpose**
   a. You defined “purpose” as such (in the survey open-ended question: What does it mean to have a purpose in your life?). Can you elaborate on your answer?
   b. Do you think you have a purpose in your life? What is your purpose in life?
   c. How do you know you’ve found your purpose? [Think about purpose as something that matters most to you and you hope to accomplish that is personally very meaningful to you.]
2. **Goal orientation**
   a. Why is this purpose so important to you? How does [your purpose] influence your life [thoughts, attitude, motivation, behavior]?
   b. How much do you care about the aim/purpose, relative to other purposes in your life?
   c. Why do you think you got involved in this particular purpose/cause rather than a different one?
   d. Are you doing anything today, this week, this month, or this year to make progress toward this purpose? If so, what?
      - If the respondent says “nothing,” probe: 1) think about it one more time; 2) have you done anything to make any progress toward your purpose/things that matter to you?
   e. How do you feel when you are engaging in [activities related to purpose that interviewees mentioned]?
3. **Processes through which purposes are discovered (Pathways to purpose)**
   a. [Could you describe] How did [the purpose] become important to you?
   b. When did it become important to you? Can you think of any particular moments or events that influenced or helped develop your purpose?
   c. What helped you find your purpose? It can be both hardships/challenges/life events as well as assets/support.
   d. Who helped you find your purpose?
   e. What were/are the obstacles? How did/do/will you overcome them? What resources/things may help you to overcome those challenges/obstacles?
4. **Future orientation**
a. What are your plans for the next few (5) years? Do you think your purpose will change in the next 5 years?
b. Picture yourself at say, 35 years old. What will you be doing? Who’ll be in your life?
c. What do you think will be important to you when you are 35 years old? Do you think your purpose will be the same (or change) when you are 35 years old?
5. **Closing**
